# Supplementary material for: Optimal treatment strategies for stage I non-small cell lung cancer in veterans with pulmonary and cardiac comorbidities
Source: PLoS One. 2021 Mar 18;16(3):e0248067. doi: 10.1371/journal.pone.0248067 (PMC7971489; doi:10.1371/journal.pone.0248067)
Supplement: S1 Table — (DOCX) [file pone.0248067.s001.docx]

| **S1 Table.** Multivariable logistic regression models of 30-day complications of lung cancer surgery among Veterans; Model 1 with COPD as a single covariate. | | | | | | | | | | |
| --- | --- | --- | --- | --- | --- | --- | --- | --- | --- | --- |
| **MODELS** | **COVARIATES**  OR (95% CI) | | | | | | | | | |
|  | **Age** (Ref: <60 yrs) | | | **CAD**  (Ref: No) | **COPD**  (Ref: No) | **Functional Status**  (Ref: No) | **Lob**  (Ref: LR^†^) | **Cancer Stage Group** (Ref: I) | |  |
|  | **60-69 yrs** | **70-79 yrs** | ≥**80 yrs** |  |  |  |  | **II** | **IIIA** |  |
| 30-day Death | NS | NS | 4.08 (1.92-8.66) | 1.68 (1.12-2.53) | 1.94 (1.31-2.87) | 3.33 (1.72-6.43) | 1.66 (1-2.75) | NS | NS |  |
| Afib | 1.99 (1.57-2.53) | 3.17 (2.48-4.06) | 5.2 (3.81-7.09) | NS | 1.16 (1.01-1.33) | NS | 1.47 (1.24-1.75) | NS | NS |  |
| Air-leak | 2.39 (1.48-3.88) | 2.41 (1.43-4.05) | NS | NS | NS | NS | 1.6 (1.08-2.37) | NS | NS |  |
| ARDS | NS | NS | NS | NS | NS | NS | NS | NS | NS |  |
| Bronchopleural Fistula | NS | NS | NS | NS | NS | NS | NS | NS | NS |  |
| Chylothorax | NS | NS | NS | NS | NS | NS | NS | NS | 3.06 (1.27-7.39) |  |
| Cerebrovascular Accident | NS | NS | 5.74 (1.43-23.07) | NS | NS | NS | NS | NS | NS |  |
| Empyema | NS | NS | NS | NS | NS | NS | NS | NS | NS |  |
| Myocardial Infarction | NS | NS | NS | 3.93 (1.81-8.57) | NS | 4.64 (1.34-16.02) | 2.44 (0.73-8.22) | NS | NS |  |
| Pneumonia | NS | 1.13 (0.86-1.49) | 1.49 (1.01-2.19) | NS | 1.98 (1.65-2.38) | 1.79 (1.16-2.76) | 1.8 (1.41-2.31) | NS | NS |  |
| Reoperation | NS | NS | NS | NS | 1.51 (1.23-1.84) | 2.12 (1.34-3.34) | 1.3 (1-1.68) | NS | NS |  |
| Sepsis | NS | NS | NS | NS | 2 (1.53-2.61) | NS | 1.76 (1.22-2.53) | NS | NS |  |
| Bleeding | 0.29 (0.1-0.86) | 0.27 (0.08-0.97) | NS | NS | 3.93 (1.28-12.08) | NS | NS | NS | NS |  |
| Post-operative infection | NS | NS | NS | NS | NS | 2.08 (1-4.33) | NS | NS | NS |  |
| Prolonged Stay | NS | 1.4 (1.11-1.77) | 2 (1.45-2.77) | NS | 1.88 (1.61-2.19) | 2 (1.39-2.89) | 1.67 (1.36-2.05) | NS | NS |  |
| Reintubation | NS | 1.47 (1.08-1.99) | 2.31 (1.55-3.44) | NS | 2.38 (1.95-2.9) | 2.27 (1.49-3.45) | 1.58 (1.22-2.04) | NS | NS |  |
| Renal Failure | NS | NS | NS | NS | 3.37 (1.58-7.17) | NS | NS | 2.11 (1.07-4.17) | NS |  |
| Respiratory Failure | NS | NS | NS | NS | 2.1 (1.54-2.86) | 2.85 (1.63-5) | NS | NS | NS |  |
| Ref = Reference  NS = Not Significant | | | | | | | | | | |
| † = Limited Resection | | | | | | | | | | |
