# Supplementary material for: Optimal treatment strategies for stage I non-small cell lung cancer in veterans with pulmonary and cardiac comorbidities
Source: PLoS One. 2021 Mar 18;16(3):e0248067. doi: 10.1371/journal.pone.0248067 (PMC7971489; doi:10.1371/journal.pone.0248067)
Supplement: S2 Table — (DOCX) [file pone.0248067.s002.docx]

| **S2 Table.** Multivariable logistic regression models of 30-day complications of lung cancer surgery among Veterans; Model 2 with GOLD stages of airway obstruction included. | | | | | | | | | | |
| --- | --- | --- | --- | --- | --- | --- | --- | --- | --- | --- |
| **MODELS** | **COVARIATES**  OR (95% CI) | | | | | | | | | |
|  | **Age** (Ref: <60 yrs) | | | **CAD**  (Ref: No) | **GOLD Stage** (Ref: Stage 0/1) | | **Functional Status**  (Ref: No) | **Lob**  (Ref: LR^†^) | **Cancer Stage Group** (Ref: I) | |
|  | **60-69 yrs** | **70-79 yrs** | ≥**80 yrs** |  | **2** | **3** |  |  | **II** | **IIIA** |
| Afib | NS | 2.6 (1.76-3.86) | 3.55 (2.14-5.88) | NS | NS | NS | NS | 1.58 (1.16-2.14) | NS | NS |
| Air-leak | 5.39 (1.27-22.86) | 8.52 (1.96-36.96) | NS | NS | NS | NS | NS | NS | NS | NS |
| ARDS | NS | NS | NS | NS | NS | NS | NS | NS | NS | NS |
| Bronchopleural Fistula | NS | NS | NS | NS | NS | NS | NS | NS | NS | NS |
| Chylothorax | NS | NS | NS | NS | NS | NS | NS | NS | NS | NS |
| Cerebrovascular Accident | NS | NS | NS | NS | NS | NS | NS | NS | NS | NS |
| Empyema | NS | NS | NS | NS | NS | NS | NS | NS | NS | NS |
| Myocardial Infarction | NS | NS | NS | NS | NS | NS | 10.03 (1.93-52.25) | NS | NS | NS |
| Pneumonia | NS | NS | NS | NS | NS | NS | NS | 1.82 (1.22-2.71) | NS | NS |
| Reoperation | NS | NS | NS | NS | NS | NS | NS | 2.44 (1.41-4.2) | 1.64 (1.11-2.43) | 1.85 (1.12-3.06) |
| Sepsis | NS | NS | NS | NS | NS | NS | NS | 3.23 (1.48-7.04) | 1.73 (1.09-2.76) | NS |
| 30-day Death | NS | NS | 7.38 (1.47-37.14) | 2.78 (1.38-5.61) | NS | NS | 4.75 (1.54-4.67) | 5.32 (1.26-22.1) | NS | NS |
| Bleeding | NS | NS | NS | 5.01 (1.12-22.48) | NS | NS | NS | NS | NS | NS |
| Post-operative infection | NS | NS | NS | NS | NS | NS | NS | NS | NS | NS |
| Prolonged Stay | NS | NS | NS | NS | NS | 1.45 (1-2.08) | NS | 2.02 (1.41-2.87) | NS | NS |
| Reintubation | NS | NS | NS | NS | NS | 1.63 (1.05-2.52) | 2.77 (1.4-5.46) | 1.67 (1.08-2.57) | NS | NS |
| Renal Failure | NS | NS | NS | NS | NS | NS | NS | NS | 4.63 (1.37-15.7) | NS |
| Respiratory Failure | NS | NS | NS | NS | NS | NS | NS | NS | NS | NS |
| Ref = Reference  NS = Not Significant | | | | | | | | | | |
| † = Limited Resection | | | | | | | | | | |
