# Supplementary material for: Optimal treatment strategies for stage I non-small cell lung cancer in veterans with pulmonary and cardiac comorbidities
Source: PLoS One. 2021 Mar 18;16(3):e0248067. doi: 10.1371/journal.pone.0248067 (PMC7971489; doi:10.1371/journal.pone.0248067)
Supplement: S3 Table — (DOCX) [file pone.0248067.s003.docx]

**S3 Table.** Multivariable regression of non-lung cancer death (n=1,853)

| **Covariates** | **Hazards Ratio**  **(95% Confidence Interval)** |
| --- | --- |
| **Age (Ref: <60 years)** |  |
| 60-69 years | 1.70 (1.10 – 2.61) |
| 70-79 years | 3.07 (2.00-4.70) |
| ≥80 years | 5.00 (3.04 – 8.20) |
| **GOLD staging (Ref: 1)** |  |
| GOLD 2 | 1.17 (0.90-1.51) |
| GOLD 3 | 1.38 (0.93 – 2.04) |
| **Coronary Artery Disease** | 1.15 (0.71 - 1.85) |
| **Tumor Histology** |  |
| Squamous Cell | Reference |
| Adenocarcinoma | 0.68 (0.53 - 0.87) |
