# Supplementary material for: Optimal treatment strategies for stage I non-small cell lung cancer in veterans with pulmonary and cardiac comorbidities
Source: PLoS One. 2021 Mar 18;16(3):e0248067. doi: 10.1371/journal.pone.0248067 (PMC7971489; doi:10.1371/journal.pone.0248067)
Supplement: S4 Table — (DOCX) [file pone.0248067.s004.docx]

| **S4 Table**. Baseline quality of life (utility) values from the Veterans Aging Cohort Status according to comorbidity (n=3,511) | | | |
| --- | --- | --- | --- |
| **Characteristic** | **Beta** | **Standard Error** | **p-value** |
| COPD | -0.021 | 0.006 | 0.0005 |
| CAD | -0.018 | 0.006 | 0.005 |
| CHF: Congestive heart failure, COPD: Chronic obstructive pulmonary disease, CKD: Chronic kidney disease; CAD: Coronary artery disease; FSI: Functional status impairment | | | |
