# Supplementary material for: Optimal treatment strategies for stage I non-small cell lung cancer in veterans with pulmonary and cardiac comorbidities
Source: PLoS One. 2021 Mar 18;16(3):e0248067. doi: 10.1371/journal.pone.0248067 (PMC7971489; doi:10.1371/journal.pone.0248067)
Supplement: S5 Table — (DOCX) [file pone.0248067.s005.docx]

| **S5 Table**. Prevalence of major toxicity following SBRT treatment (n=386) | | |
| --- | --- | --- |
| **SBRT Toxicity** | **Proportion** | **95% CI** |
| Esophagitis | 0.77% | 0.16%-2.20% |
| Pneumonitis | 0.52% | 0.06%-1.90% |
| Hemoptysis | 0.26% | 0.01%-1.40% |
| Any Major Toxicity | 1.55% | 0.57%-3.40% |
| SBRT: Stereotactic body radiotherapy; CI: Confidence Interval | | |
