# Supplementary material for: Optimal treatment strategies for stage I non-small cell lung cancer in veterans with pulmonary and cardiac comorbidities
Source: PLoS One. 2021 Mar 18;16(3):e0248067. doi: 10.1371/journal.pone.0248067 (PMC7971489; doi:10.1371/journal.pone.0248067)
Supplement: S6 Table — A. Estimates of quality-adjusted life year gains for different stage I NSCLC treatment options in veterans for patients with no COPD or GOLD stage 1. B. Estimates of quality-adjusted life year gains for different stage I NSCLC treatment options in veterans for patients with GOLD stage 2. C. Estimates of quality-adjusted life year gains for different stage I NSCLC treatment options in veterans for patients with GOLD stage 3. (DOCX) [file pone.0248067.s006.docx]

| **S6 Table. A.** Estimates of Quality-Adjusted Life Year Gains for Different Stage I NSCLC Treatment Options in Veterans for Patients with No COPD or GOLD Stage 1. | | | | | | | | |
| --- | --- | --- | --- | --- | --- | --- | --- | --- |
| **Size / Histologic Subtype** | **Age Group (Years)** | | **Quality-Adjusted Life Years Gained** | | | | | |
|  |  |  | **Coronary Artery Disease** | | | **No Coronary Artery Disease** | | |
|  |  |  | **Lob** | **LR** | **SBRT** | **Lob** | **LR** | **SBRT** |
|  | | |  | | |  |  |  |
| **<1 cm Adenocarcinoma** | | <60 | 19.2 | 18.1 | 17.9 | 19.5 | 18.1 | 17.9 |
|  |  | 60-69 | 15.5 | 14.9 | 14.8 | 15.7 | 14.9 | 14.8 |
|  |  | 70-79 | 10.9 | 10.6 | 10.5 | 11.0 | 10.6 | 10.5 |
|  |  | ≥80 | 6.5 | 7.1 | 7.1 | 6.8 | 7.1 | 7.1 |
| **1-2 cm Adenocarcinoma** | | <60 | 19.2 | 17.0 | 16.3 | 19.5 | 17.0 | 16.3 |
|  |  | 60-69 | 15.5 | 14.2 | 13.8 | 15.7 | 14.2 | 13.8 |
|  |  | 70-79 | 10.9 | 10.2 | 9.9 | 11.0 | 10.2 | 9.9 |
|  |  | ≥80 | 6.5 | 6.9 | 6.8 | 6.8 | 6.9 | 6.8 |
| **2-3 cm Adenocarcinoma** | | <60 | 19.2 | 17.0 | 15.7 | 19.5 | 17.0 | 15.7 |
|  |  | 60-69 | 15.5 | 14.2 | 13.4 | 15.7 | 14.2 | 13.4 |
|  |  | 70-79 | 10.9 | 10.2 | 9.7 | 11.0 | 10.2 | 9.7 |
|  |  | ≥80 | 6.5 | 6.9 | 6.7 | 6.8 | 6.9 | 6.7 |
| **>3cm Adenocarcinoma** | | <60 | 19.2 | 17.0 | 14.3 | 19.5 | 17.0 | 14.3 |
|  |  | 60-69 | 15.5 | 14.2 | 12.4 | 15.7 | 14.2 | 12.4 |
|  |  | 70-79 | 10.9 | 10.2 | 9.1 | 11.0 | 10.2 | 9.1 |
|  |  | ≥80 | 6.5 | 6.9 | 6.5 | 6.8 | 6.9 | 6.5 |
| **<1 cm Squamous Cell Carcinoma** | | <60 | 17.2 | 16.2 | 16.0 | 17.5 | 16.2 | 16.0 |
|  |  | 60-69 | 13.7 | 13.2 | 13.1 | 13.9 | 13.2 | 13.1 |
|  |  | 70-79 | 9.6 | 9.3 | 9.3 | 9.7 | 9.3 | 9.3 |
|  |  | ≥80 | 6.0 | 6.5 | 6.5 | 6.2 | 6.5 | 6.5 |
| **1-2 cm Squamous Cell Carcinoma** | | <60 | 17.2 | 15.3 | 14.6 | 17.5 | 15.3 | 14.6 |
|  |  | 60-69 | 13.7 | 12.6 | 12.2 | 13.9 | 12.6 | 12.2 |
|  |  | 70-79 | 9.6 | 9.0 | 8.8 | 9.7 | 9.0 | 8.8 |
|  |  | ≥80 | 6.0 | 6.3 | 6.3 | 6.2 | 6.3 | 6.3 |
| **2-3 cm Squamous Cell Carcinoma** | | <60 | 17.2 | 15.3 | 14.1 | 17.5 | 15.3 | 14.1 |
|  |  | 60-69 | 13.7 | 12.6 | 11.9 | 13.9 | 12.6 | 11.9 |
|  |  | 70-79 | 9.6 | 9.0 | 8.6 | 9.7 | 9.0 | 8.6 |
|  |  | ≥80 | 6.0 | 6.3 | 6.2 | 6.2 | 6.3 | 6.2 |
| **>3 cm Squamous Cell Carcinoma** | | <60 | 17.2 | 15.3 | 12.8 | 17.5 | 15.3 | 12.8 |
|  |  | 60-69 | 13.7 | 12.6 | 11.0 | 13.9 | 12.6 | 11.0 |
|  |  | 70-79 | 9.6 | 9.0 | 8.1 | 9.7 | 9.0 | 8.1 |
|  |  | ≥80 | 6.0 | 6.3 | 5.9 | 6.2 | 6.3 | 5.9 |

| **S6 Table. B.** Estimates of Quality-Adjusted Life Year Gains for Different Stage I NSCLC Treatment Options in Veterans for Patients with GOLD Stage 2. | | | | | | | |
| --- | --- | --- | --- | --- | --- | --- | --- |
| **Size / Histologic Subtype** | **Age Group (Years)** | **Quality-Adjusted Life Years Gained** | | | | | |
|  |  | **Coronary Artery Disease** | | | **No Coronary Artery Disease** | | |
|  |  | **Lob** | **LR** | **SBRT** | **Lob** | **LR** | **SBRT** |
|  | |  | | |  |  |  |
| **<1 cm Adenocarcinoma** | <60 | 18.3 | 17.1 | 16.9 | 18.6 | 17.1 | 16.9 |
|  | 60-69 | 14.9 | 14.3 | 14.2 | 15.1 | 14.3 | 14.2 |
|  | 70-79 | 10.5 | 10.2 | 10.2 | 10.7 | 10.2 | 10.2 |
|  | ≥80 | 6.4 | 6.9 | 6.9 | 6.7 | 6.9 | 6.9 |
| **1-2 cm Adenocarcinoma** | <60 | 18.3 | 16.0 | 15.4 | 18.6 | 16.0 | 15.4 |
|  | 60-69 | 14.9 | 13.6 | 13.1 | 15.1 | 13.6 | 13.1 |
|  | 70-79 | 10.5 | 9.8 | 9.5 | 10.7 | 9.8 | 9.5 |
|  | ≥80 | 6.4 | 6.8 | 6.7 | 6.7 | 6.8 | 6.7 |
| **2-3 cm Adenocarcinoma** | <60 | 18.3 | 16.0 | 14.8 | 18.6 | 16.0 | 14.8 |
|  | 60-69 | 14.9 | 13.6 | 12.7 | 15.1 | 13.6 | 12.7 |
|  | 70-79 | 10.5 | 9.8 | 9.3 | 10.7 | 9.8 | 9.3 |
|  | ≥80 | 6.4 | 6.8 | 6.6 | 6.7 | 6.8 | 6.6 |
| **>3cm Adenocarcinoma** | <60 | 18.3 | 16.0 | 13.3 | 18.6 | 16.0 | 13.3 |
|  | 60-69 | 14.9 | 13.6 | 11.7 | 15.1 | 13.6 | 11.7 |
|  | 70-79 | 10.5 | 9.8 | 8.7 | 10.7 | 9.8 | 8.7 |
|  | ≥80 | 6.4 | 6.8 | 6.3 | 6.7 | 6.8 | 6.3 |
| **<1 cm Squamous Cell Carcinoma** | <60 | 16.4 | 15.3 | 15.2 | 16.6 | 15.3 | 15.2 |
|  | 60-69 | 13.2 | 12.6 | 12.6 | 13.4 | 12.6 | 12.6 |
|  | 70-79 | 9.3 | 9.0 | 9.0 | 9.4 | 9.0 | 9.0 |
|  | ≥80 | 5.8 | 6.3 | 6.3 | 6.1 | 6.3 | 6.3 |
| **1-2 cm Squamous Cell Carcinoma** | <60 | 16.4 | 14.4 | 13.8 | 16.6 | 14.4 | 13.8 |
|  | 60-69 | 13.2 | 12.0 | 11.6 | 13.4 | 12.0 | 11.6 |
|  | 70-79 | 9.3 | 8.7 | 8.5 | 9.4 | 8.7 | 8.5 |
|  | ≥80 | 5.8 | 6.2 | 6.1 | 6.1 | 6.2 | 6.1 |
| **2-3 cm Squamous Cell Carcinoma** | <60 | 16.4 | 14.4 | 13.3 | 16.6 | 14.4 | 13.3 |
|  | 60-69 | 13.2 | 12.0 | 11.3 | 13.4 | 12.0 | 11.3 |
|  | 70-79 | 9.3 | 8.7 | 8.2 | 9.4 | 8.7 | 8.2 |
|  | ≥80 | 5.8 | 6.2 | 6.0 | 6.1 | 6.2 | 6.0 |
| **>3 cm Squamous Cell Carcinoma** | <60 | 16.4 | 14.4 | 12.0 | 16.6 | 14.4 | 12.0 |
|  | 60-69 | 13.2 | 12.0 | 10.4 | 13.4 | 12.0 | 10.4 |
|  | 70-79 | 9.3 | 8.7 | 7.7 | 9.4 | 8.7 | 7.7 |
|  | ≥80 | 5.8 | 6.2 | 5.8 | 6.1 | 6.2 | 5.8 |

| **S6 Table. C**. Estimates of Quality-Adjusted Life Year Gains for Different Stage I NSCLC Treatment Options in Veterans for Patients with GOLD Stage 3. | | | | | | | |
| --- | --- | --- | --- | --- | --- | --- | --- |
| **Size / Histologic Subtype** | **Age Group (Years)** | **Quality-Adjusted Life Years Gained** | | | | | |
|  |  | **Coronary Artery Disease** | | | **No Coronary Artery Disease** | | |
|  |  | **Lob** | **LR** | **SBRT** | **Lob** | **LR** | **SBRT** |
|  | |  | | |  |  |  |
| **<1 cm Adenocarcinoma** | <60 | 15.7 | 14.5 | 14.4 | 15.9 | 14.5 | 14.4 |
|  | 60-69 | 12.9 | 12.2 | 12.1 | 13.0 | 12.2 | 12.1 |
|  | 70-79 | 9.1 | 8.8 | 8.8 | 9.2 | 8.8 | 8.8 |
|  | ≥80 | 5.8 | 6.3 | 6.3 | 6.0 | 6.3 | 6.3 |
| **1-2 cm Adenocarcinoma** | <60 | 15.7 | 13.6 | 13.0 | 15.9 | 13.6 | 13.0 |
|  | 60-69 | 12.9 | 11.6 | 11.1 | 13.0 | 11.6 | 11.1 |
|  | 70-79 | 9.1 | 8.4 | 8.2 | 9.2 | 8.4 | 8.2 |
|  | ≥80 | 5.8 | 6.1 | 6.0 | 6.0 | 6.1 | 6.0 |
| **2-3 cm Adenocarcinoma** | <60 | 15.7 | 13.6 | 12.4 | 15.9 | 13.6 | 12.4 |
|  | 60-69 | 12.9 | 11.6 | 10.8 | 13.0 | 11.6 | 10.8 |
|  | 70-79 | 9.1 | 8.4 | 8.0 | 9.2 | 8.4 | 8.0 |
|  | ≥80 | 5.8 | 6.1 | 5.9 | 6.0 | 6.1 | 5.9 |
| **>3cm Adenocarcinoma** | <60 | 15.7 | 13.6 | 11.2 | 15.9 | 13.6 | 11.2 |
|  | 60-69 | 12.9 | 11.6 | 9.9 | 13.0 | 11.6 | 9.9 |
|  | 70-79 | 9,1 | 8.4 | 7.4 | 9.2 | 8.4 | 7.4 |
|  | ≥80 | 5.8 | 6.1 | 5.6 | 6.0 | 6.1 | 5.6 |
| **<1 cm Squamous Cell Carcinoma** | <60 | 13.8 | 12.8 | 12.7 | 14.0 | 12.8 | 12.7 |
|  | 60-69 | 11.1 | 10.6 | 10.5 | 11.3 | 10.6 | 10.5 |
|  | 70-79 | 7.8 | 7.6 | 7.6 | 8.0 | 7.6 | 7.6 |
|  | ≥80 | 5.2 | 5.6 | 5.6 | 5.4 | 5.6 | 5.6 |
| **1-2 cm Squamous Cell Carcinoma** | <60 | 13.8 | 12.0 | 11.5 | 14.0 | 12.0 | 11.5 |
|  | 60-69 | 11.1 | 10.1 | 9.7 | 11.3 | 10.1 | 9.7 |
|  | 70-79 | 7.8 | 7.3 | 7.1 | 8.0 | 7.3 | 7.1 |
|  | ≥80 | 5.2 | 5.5 | 5.4 | 5.4 | 5.5 | 5.4 |
| **2-3 cm Squamous Cell Carcinoma** | <60 | 13.8 | 12.0 | 11.0 | 14.0 | 12.0 | 11.0 |
|  | 60-69 | 11.1 | 10.1 | 9.4 | 11.3 | 10.1 | 9.4 |
|  | 70-79 | 7.8 | 7.3 | 7.0 | 8.0 | 7.3 | 7.0 |
|  | ≥80 | 5.2 | 5.5 | 5.3 | 5.4 | 5.5 | 5.3 |
| **>3 cm Squamous Cell Carcinoma** | <60 | 13.8 | 12.0 | 9.9 | 14.0 | 12.0 | 9.9 |
|  | 60-69 | 11.1 | 10.1 | 8.7 | 11.3 | 10.1 | 8.7 |
|  | 70-79 | 7.8 | 7.3 | 6.5 | 8.0 | 7.3 | 6.5 |
|  | ≥80 | 5.2 | 5.5 | 5.1 | 5.4 | 5.5 | 5.1 |
