# Supplementary figures and images for: Optimal treatment strategies for stage I non-small cell lung cancer in veterans with pulmonary and cardiac comorbidities
Source: PLoS One. 2021 Mar 18;16(3):e0248067. doi: 10.1371/journal.pone.0248067 (PMC7971489; doi:10.1371/journal.pone.0248067)

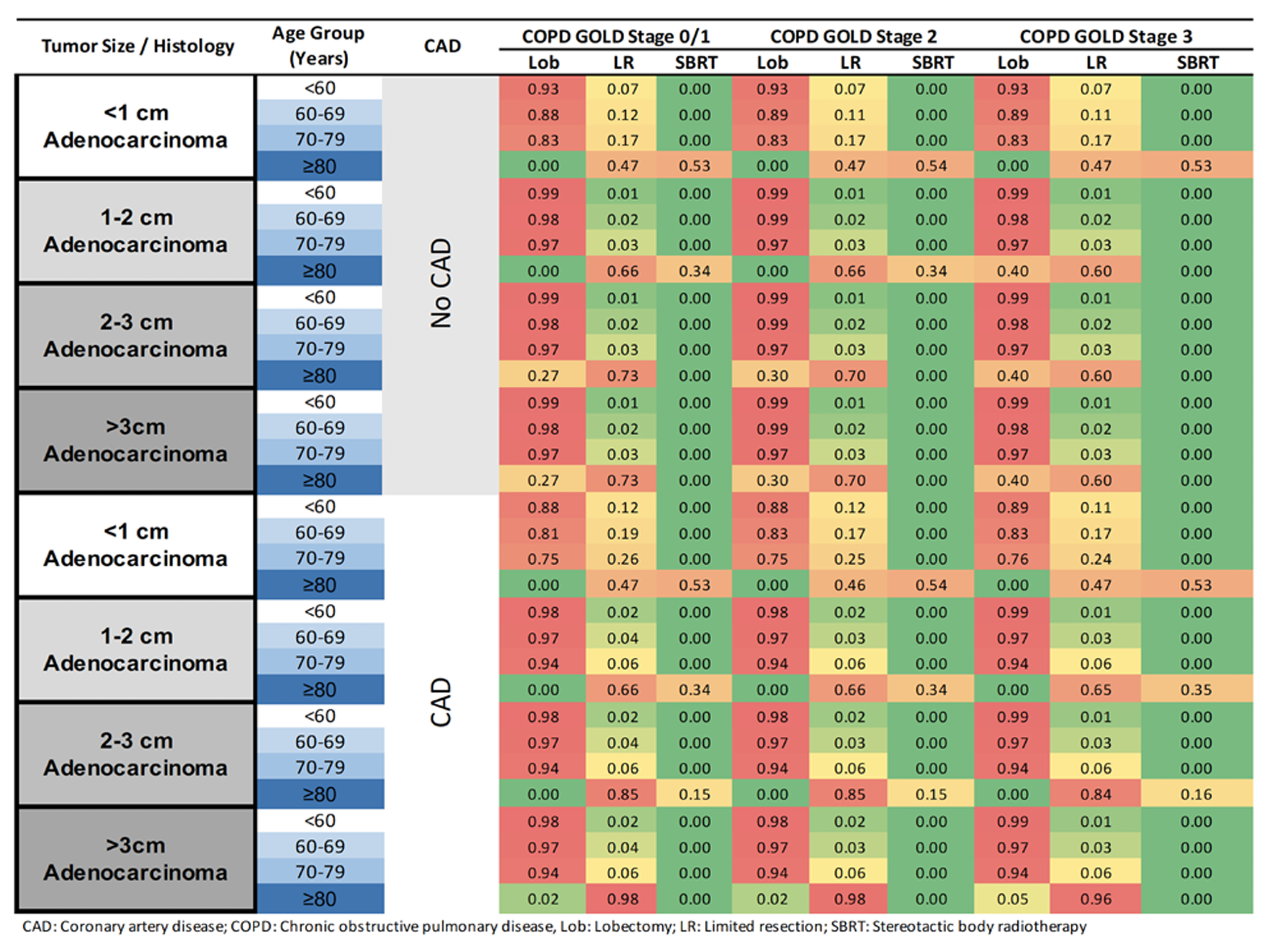

Supplement: S1 Fig — Probabilities represent the proportion of simulations where a treatment strategy was the optimal modality for maximizing QALYs gained. (TIF) [file pone.0248067.s007.tif]

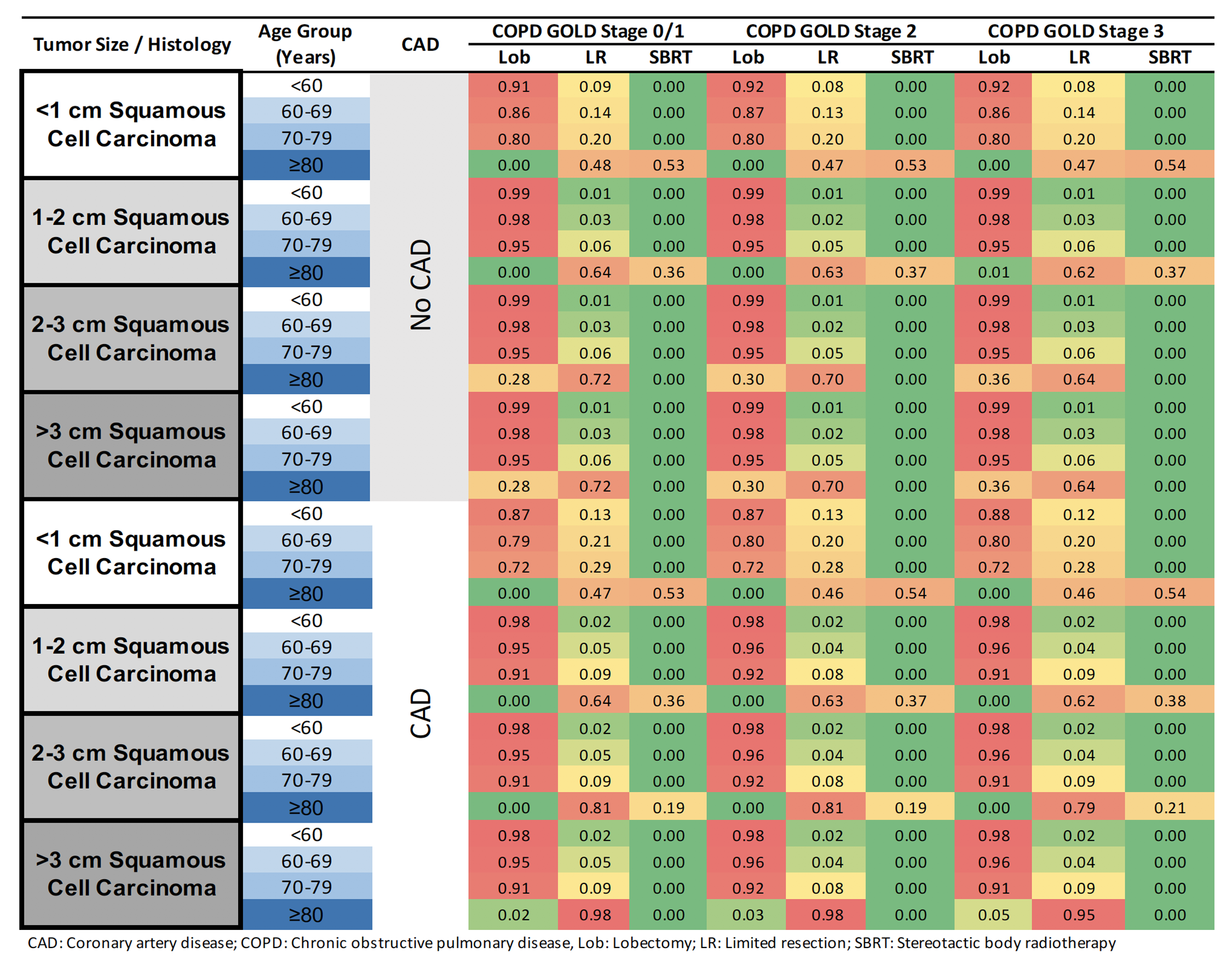

Supplement: S2 Fig — (TIF) [file pone.0248067.s008.tif]
